# Differential Expression and Localization of EHBP1L1 during the First Wave of Rat Spermatogenesis Suggest Its Involvement in Acrosome Biogenesis †

**DOI:** 10.3390/biomedicines10010181

**Published:** 2022-01-16

**Authors:** Massimo Venditti, Sergio Minucci

**Affiliations:** Dipartimento di Medicina Sperimentale, Università degli Studi della Campania “Luigi Vanvitelli”, Via Costantinopoli 16, 80138 Napoli, Italy

**Keywords:** EHBP1L1, acrosome, rat testis, spermatogenesis, Golgi apparatus, cytoskeleton

## Abstract

The identification and characterization of new proteins involved in spermatogenesis is fundamental, considering that good-quality gametes are basic in ensuring proper reproduction. Here, we further analyzed the temporal and spatial localization during the first spermatogenic wave of rat testis of EHBP1L1, which is involved in vesicular trafficking due to the CH and bMERB domains, which bind to actin and Rab8/10, respectively. Western blot and immunofluorescence analyses showed that EHBP1L1 protein expression started at 21 days post-partum (dpp) concomitantly with the appearance of primary spermatocytes (I SPC). In subsequent stages, EHBP1L1 specifically localized together with actin in the perinuclear cytoplasm close to the acrosomal and Golgian regions of spermatids (SPT) during the different phases of acrosome biogenesis (AB). Moreover, it was completely absent in elongated SPT and in mature spermatozoa, suggesting that its role was completed in previous stages. The combined data, also supported by our previous report demonstrating that *EHBP1L1* mRNA was expressed by primary (I) and secondary (II) SPC, lead us to hypothesize its specific role during AB. Although these results are suggestive, further studies are needed to better clarify the underlying molecular mechanisms of AB, with the aim to use EHBP1L1 as a potential new marker for spermatogenesis.

## 1. Introduction

Reproductive success is one of the main purposes of a species that ensures its perpetuation and genetic variability to reach proper fitness. Therefore, the production and differentiation of good-quality gametes is extremely necessary. Of note, the acrosome reaction represents one of the key events that occurs during fertilization that is needed not only for the species-specific recognition between male and female gametes, but also to permit the penetration of the male pronucleus into the oocyte, prior to the digestion of the zona pellucida, to form the zygote [1,2].

Moreover, spermatozoa (SPZ) possessing a normal acrosome are favored in fertilization; it has been demonstrated that intra-cytoplasmic insemination experiments using SPZ with an abnormal acrosome resulted in unsuccessful fertilization [3,4,5], highlighting that this organelle is fundamental for reproductive efficacy. Consequently, acrosome biogenesis (AB) is also extremely important. Acrosome formation that is comprehensive of four characteristic phases (the Golgi, the cap, the acrosome and the maturation phases), takes place during spermiogenesis, the differentiative process that leads round spermatids (rSPT) to form elongated SPT (eSPT); finally, to obtain mature and qualitatively functional SPZ, each step must be realized and controlled in a precise manner by genes that are activated and/or repressed in specific cells and stages [6].

In the Golgi phase, acrosomal components are accumulated into many pro-acrosomic vesicles budding from the trans-Golgi network and are then transported to the concave region of the nuclear surface, where they fuse into a single granule [7,8]. During the cap phase, the developing acrosome flattens itself onto the nucleus, forming a cap-like structure; simultaneously, the Golgi apparatus relocates to the forming neck region. In the acrosome phase, the acrosome starts condensing, while in the maturation phase, the completely formed acrosome spreads over the nuclear surface, covering it almost entirely [8]. Moreover, for a proper AB, the precise coordination between cytoplasmic organelles (endoplasmic reticulum, Golgi apparatus and nucleus) and cytoplasmic cytoskeletal structures (such as acroplaxome and manchette, which help acrosome and nucleus to acquire their definitive shape and location) is needed [8]. However, the complexity of this scenario is reflected by the fact that, apart from the one involving vesicular trafficking originating from the Golgi apparatus, other mechanisms may contribute to AB since many authors have proposed the acrosome as a specialized lysosome and/or a secretory granule, as both the biosynthetic and endocytic pathways concur to its biogenesis [7,8,9,10]. Many proteins notoriously involved in the endocytic pathway in somatic cells have been identified in biosynthetic/anterograde vesicular trafficking in the AB that occurs during spermiogenesis [7,8,9,10]. Thus, the identification of spermatogenic-related genes, and those involved in the AB, is essential to clarify and obtain more information on the mechanisms underlying reproductive activity.

In a previous paper, we isolated, for the first time in the rat testis, a partial cDNA clone encoding for EH domain binding protein 1-like 1 (*EHBP1L1*; previously named *Tangerin*), a protein possessing a single calponin homology (CH) domain that notoriously has been correlated with signaling and cytoskeletal actin-related processes [11]. Remarkably, in situ hybridization of testis sections and RT-PCR experiments performed on RNA extracted from different testicular cell types-enriched fractions showed a specific localization and expression of *EHBP1L1* in primary (I) and secondary (II) spermatocytes (SPC); contrarily, no evidence was observed in spermatogonia (SGP), SPT and SPZ [10]. Therefore, we hypothesized the involvement of *EHBP1L1* in AB during rat spermiogenesis since different papers described this protein in polarized somatic cells as an adapter between vesicular transport from the trans-Golgi network and the apical plasma membrane (due to its ability to form, on the vesicle surface, a multiprotein complex together with Rab8/10, Bin1/amphiphysin II and dynamin) [12,13,14] and actin microfilaments (due to the CH domain) [15].

Thus, to confirm this assumption and to amplify the knowledge about EHBP1L1 and its biological role in spermatogenesis, in this paper, we evaluated its protein expression and localization together with acrosome and actin. The analysis was performed during the first wave of rat spermatogenesis, which represents a good model that, due to the gradual appearance and differentiation of the several cell types composing the seminiferous epithelium, allows for the obtainment of localization profiles—cell- and stage-specific—of genes and proteins involved in this process.

## 2. Materials and Methods

### 2.1. Animal Care, Tissue Extraction, and Collection of Rat Spermatozoa

Male Sprague–Dawley rats (*Rattus norvegicus*) were kept in specific conditions (12D:12L), and they were provided with standard food and water ad libitum. Animals at different development stages (7, 14, 21, 28, 35, 42, 60 days postpartum (dpp); *n* = 3 per stage) were sacrificed under anesthesia with ketamine (100 mg/kg i.p.) in accordance with national and local guidelines covering experimental animals. For each animal, testes were dissected, the left one was fixed in Bouin’s fluid and embedded in paraffin for histological analysis, and the right one was quickly frozen by immersion in liquid nitrogen and stored at −80 °C until protein extraction. In addition, epididymides were dissected from adult rats and minced in phosphate-buffer saline (PBS; 13.6 mM NaCl; 2.68 mM KCl; 8.08 mM Na_2_HPO_4_; 18.4 mM KH_2_PO_4_; 0.9 mM CaCl_2_; 0.5 mM MgCl_2_; pH 7.4) to allow the SPZ flow out from the ducts. Then, the fluid samples were filtered and examined under a light microscope to exclude contamination by other cell types. Next, aliquots were spotted and air-dried on slides and stored at −20 °C; the remaining samples were centrifuged at 1000× *g* for 15 min at 4 °C and stored at −80 °C until protein extraction. The experimental procedure was approved by the Ethics Committee for Research in life science and health of the Higher Institute of Biotechnology of Monastir, Tunisia (CER-SVS/ISBM—protocol 022/2020) and was carried out according to the UNESCO Recommendation Concerning Science and Scientific Research (1974, 2017).

### 2.2. Isolation of Germinal Cells by Centrifugal Elutriation

Testes from two adult rats were decapsulated, resuspended in 10 mL of Dulbecco’s minimal essential medium (DMEM) and treated with collagenase type I treatment (0.25 mg/mL; Sigma-Aldrich Corp., Milan, Italy) to obtain seminiferous tubules free of interstitial tissues. Tubules were then incubated at 37 °C for 60 min in DMEM containing collagenase (0.25 mg/mL), DNase I (0.075 mg/mL; Sigma-Aldrich Corp., Milan, Italy), and 0.5% bovine serum albumin (BSA; Sigma-Aldrich Corp., Milan, Italy). After incubation, the cell suspension was centrifuged for 10 min at 1200× *g*. Propidium iodide was added to aliquots of the pellet and subjected to cytofluorimetry analysis in a Becton–Dickinson cytofluorimeter. Total germinal cells were resuspended in DMEM, in the presence of 0.1 mg/mL DNase I and 0.5% BSA, and enriched fractions of I and II SPC and SPT were obtained using centrifugal elutriation using a JE-6 Beckman elutriator rotor, as described in our previous work [11].

### 2.3. Collection of Human Spermatozoa

Human sperm samples (*n* = 5) were collected by masturbation from donors at the “Centre for Assisted Fertilization” in Naples (Via Tasso, 480, 80123, Naples, Italy), and the chemical, physical, and spermatic parameters were evaluated in accordance with the World Health Organization (WHO) guidelines to verify the good quality of the samples [16]. The samples were centrifuged at 800× *g* for 10 min; the supernatant was removed, and the pellet was washed and resuspended in PBS. The suspensions were examined under a light microscope, and aliquots were spotted and air-dried on slides and then stored at −20 °C, while the remaining parts were centrifuged at 1000× *g* for 15 min at 4 °C and stored at −80 °C until protein extraction. The study was conducted according to the guidelines of the Declaration of Helsinki and approved by the Ethics Committee of “Università degli Studi della Campania Luigi Vanvitelli” (protocol number 206, approved on 15 April 2019).

### 2.4. Preparation of Total Protein Extracts and Western Blot Analysis

The testes, the enriched cell fractions (I and II SPC and SPT) and the SPZ (from rat and human) samples were lysed in RIPA buffer (1% NP-40, 0.1% SDS, 1 mM sodium orthovanadate, 0.5% sodium deoxycholate in PBS) in the presence of protease inhibitors (4 μg/μL of leupeptin, aprotinin, pepstatin A, chymostatin, PMSF). The homogenates were sonicated twice by three strokes (20 Hz for 20 s each); after centrifugation for 30 min at 10,000 g at 4 °C, the resulting supernatants were stored at −80 °C. Next, 40 μg of proteins were separated by 9% SDS-PAGE and transferred to Hybond-P polyvinylidene difluoride membranes (Amersham Pharmacia Biotech., Buckinghamshire, UK) at 280 mA for 2.5 h at 4 °C [17]. The filters were treated with blocking solution (5% skim milk in TBS (10 mM Tris–HCl pH 7.6, 150 mM NaCl) supplemented with 0.25% Tween-20 (Sigma-Aldrich Corp., Milan, Italy)) before the addition of anti-EHBP1L1 (#orb183327; Biorbyt, Cambridge, UK), or anti-β-Actin (#E-AB-20031; Elabscience Biotechnology, Wuhan, China) antibodies diluted 1:2000 and 1:10,000 in the blocking solution, respectively, and incubated overnight at 4 °C. After three washes in TBST (TBS including 0.25% Tween-20), the filters were incubated with horseradish peroxidase-conjugated antirabbit IgG (#AP307P; Sigma-Aldrich Corp., Milan, Italy) for the rabbit anti-EHBP1L1 antibody, or anti-mouse IgG (#AP130P; Sigma-Aldrich Corp., Milan, Italy) for the mouse anti- β-Actin antibody, both diluted 1:10,000 in the blocking solution. Then, the membranes were washed again three times in TBST and the immunocomplexes were revealed using the enhanced chemiluminescence (ECL) Western blotting detection system (Amersham Pharmacia Biotech., Buckinghamshire, UK). ImageJ software (version 1.53 g; NIH, Bethesda, MD, USA) was used to analyze all bands. Each Western blot experiment was performed in triplicate.

### 2.5. Immunofluorescence Analysis on Testis

For EHBP1L1 colocalization with the acrosome system, ACTIN, and the Golgi apparatus, 5 µm serial sections were processed as described by Venditti et al. [18]. Antigen retrieval was performed by pressure immersing the slides in 0.01 M citrate buffer (pH 6.0) for 3 min. To reduce autofluorescence background, slides were quenched with 0.3 M glycine in PBS for 30 min. Then, tissue permeabilization was obtained by incubating the slides with 0.1% (*v/v*) Triton X-100 in PBS for 30 min. Later, nonspecific binding sites were blocked with 5% BSA and normal goat serum diluted 1:5 in PBS before the addition of anti-EHBP1L1, or anti-β-Actin or anti-GOLGB1 (#HPA011008, Sigma-Aldrich, Milan, Italy) antibodies diluted 1:100 in the blocking solution for overnight incubation at 4 °C. After three washes in PBS, slides were incubated for 1 hr with the appropriate secondary antibody (#A32731; Alexa Fluor 488, Thermo Fisher Scientific, Waltham, MA, USA; #SAB4600082; Anti-Mouse IgG 568, Sigma-Aldrich, Milan, Italy) diluted 1:500 in the blocking mixture and with PNA lectin (#L32458; Alexa Fluor 568, Thermo Fisher Scientific, Waltham, MA, USA) diluted 1:50. The slides were washed again three times in PBS and then mounted with Vectashield + DAPI (Vector Laboratories, Peterborought, UK) for nuclear staining and were observed under an optical microscope (Leica DM 5000 B + CTR 5000; Leica Microsystems, Wetzlar, Germany) with UV lamp; images were viewed and saved with IM 1000 software (version 4.7.0; Leica Microsystems, Wetzlar, Germany). Photographs were taken using the Leica DFC320 R2 digital camera. As anti-EHBP1L1 and anti-GOLGB1 primary antibodies were both raised in rabbits, subsequent serial sections were used and incubated with anti-EHBP1L1 or with anti-GOLGB1; finally, to obtain the merged image of their co-localization, the GOLGB1 fluorescent green signal was converted to red using IM 1000 software.

### 2.6. Immunofluorescence Analysis on SPZ

To determine EHBP1L1 localization in rat and human SPZ, the samples were first fixed in 4% paraformaldehyde in PBS for 10 min and then washed twice in PBS. The slides were incubated with 0.1% (*v/v*) Triton X-100 in PBS for 30 min. Later, nonspecific binding sites were blocked with 5% BSA and normal goat serum diluted 1:5 in PBS before the addition of the anti-EHBP1L1 primary antibody, as described above, and overnight incubation at 4 °C. After three washes in PBS, slides were incubated for 1 hr with the secondary antibody (#A32731; Alexa Fluor 488, Thermo Fisher Scientific, Waltham, MA, USA) diluted 1:500 in the blocking mixture and with PNA lectin diluted 1:50. The slides were washed again in PBS and then mounted with Vectashield + DAPI (Vector Laboratories, Peterborought, UK) for nuclear staining and were then observed under an optical microscope (Leica DM 5000 B + CTR 5000; Leica Microsystems, Wetzlar, Germany) with UV Lamp; images were viewed and saved with IM 1000 software. Photographs were taken using the Leica DFC320 R2 digital camera.

### 2.7. Statistical Analysis

Data were reported as means ± standard error (SEM). Differences between the groups were considered statistically significant at *p* < 0.05. Analyses were performed using one-way ANOVA, and Tukey’s post hoc t test was applied when appropriate with Prism 5.0, GraphPad Software (San Diego, CA, USA).

## 3. Results

### 3.1. EHBP1L1 Protein Level during the First Wave of Rat Spermatogenesis

**Figure 1:**
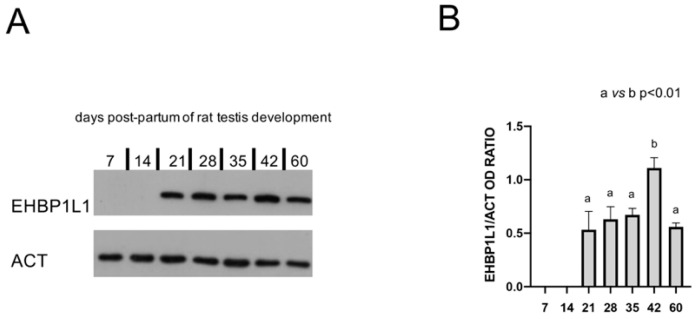
EHBP1L1 protein level during the first wave of rat spermatogenesis. (**A**): Western blot analysis showing the expression of EHBP1L1 (164 kDa, top section) and ACTIN (42 kDa, bottom section) during rat testis post-natal development at 7, 14, 21, 28, 35, 42, and 60 days postpartum (dpp). (**B**): Histogram showing the relative expression levels of EHBP1L1 in all the analyzed samples. Data were normalized with ACTIN and reported as EHBP1L1/ACT OD ratio. All data represent the mean ± SEM. a vs. b: *p* < 0.01. Western blot experiment was performed in triplicate.

The protein level of EHBP1L1 during the postnatal development of the testis was assessed by Western Blot analysis on total extracts from some of the most representative time points during the first wave of spermatogenesis [19,20]: 7 dpp (transition of gonocytes from the tubule lumen toward the base), 14 dpp (proliferation of SPG, before meiosis), 21 dpp (presence of SPC, which start meiosis), 28 dpp (conclusion of the first meiosis and appearance of rSPT), 35 dpp (presence of newly formed eSPT in spermiogenesis), 42 dpp (final steps of spermiogenesis), and 60 dpp (mature testis; presence of SPZ and of all the characteristic germ cell associations). A specific band of 164 kDa was detected for EHBP1L1 in all the analyzed samples, except for 7 and 14 dpp (Figure 1A). Bioinformatic analysis evidenced the presence of several rat EHBP1L1 isoforms possessing different molecular weight sizes (ranging from 84 to 180 kDa). Although the used antibody is able to recognize all isoforms (Figure S1), finding only a unique band suggests that only one isoform is expressed in the rat testis at the tested dpps, which likely corresponds to EHBP1L1 isoform 2 (NCBI Accession: NP_001123469.1 GI: 194018463) since all the other isoforms present lower predicted nonglycosilated molecular weights.

Moreover, the analysis of the relative protein level, reported as EHBP1L1/ACT OD ratio (Figure 1B), revealed the highest peak at 42 dpp, as compared to all the others (*p* < 0.01).

### 3.2. EHBP1L1 Localization during the First Wave of Rat Spermatogenesis

**Figure 2:**
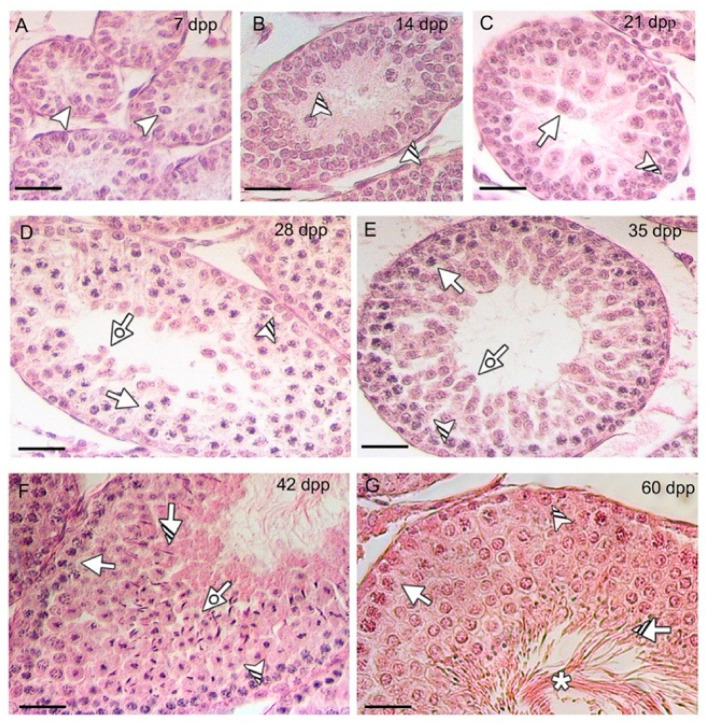
Histology and staging of the developing rat testis. Hematoxylin-eosin staining of tissue sections at 7 (**A**), 14 (**B**), 21 (**C**), 28 (**D**), 35 (**E**), 42 (**F**), 60 (**G**) dpp, in which the most representative cell types are highlighted. Scale bars represent 20 μm. Arrowhead: gonocytes; striped arrowhead: spermatogonia (SPG); arrow: spermatocyte (SPC); dotted arrow: round spermatid (rSPT); striped arrow: elongated spermatid (eSPT); asterisk: spermatozoa (SPZ) tails.

First, testicular cell-types and staging were checked by performing a hematoxylin-eosin staining on sections of the rat testis at the same time points as described in the previous paragraph (Figure 2).

**Figure 3:**
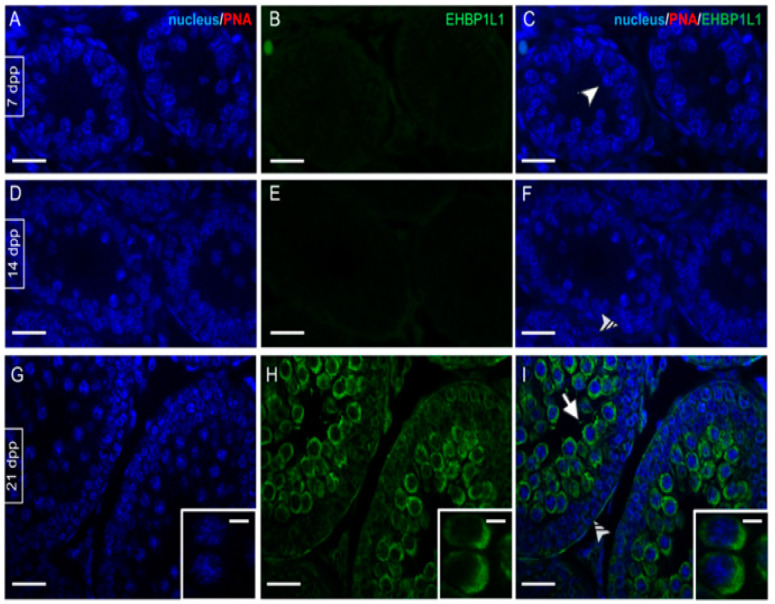
Localization of EHBP1L1 during the first wave of rat spermatogenesis (7–21 dpp). (**A**,**D**,**G**) DAPI-fluorescent nuclear staining (blue) and PNA lectin acrosome staining (red). (**B**,**E**,**H**) EHBP1L1 fluorescence (green). (**C**,**F**,**I**) Merged fluorescent channels (blue/red/green). (**A**–**C**) 7 dpp testis; (**D**–**F**) 14 dpp; (**G**–**I**) 21 dpp. Scale bars represent 20 μm, except for the insets, where they represent 10 μm. Arrowhead: gonocytes; striped arrowhead: SPG; arrow: I SPC.

**Figure 4:**
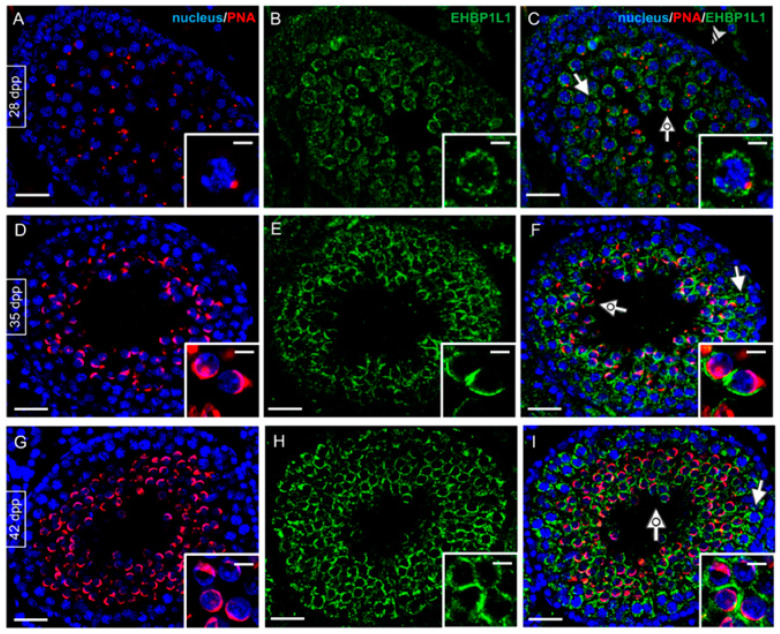
Localization of EHBP1L1 during the first wave of rat spermatogenesis (28–42 dpp). (**A**,**D**,**G**) DAPI-fluorescent nuclear staining (blue) and PNA lectin acrosome staining (red). (**B**,**E**,**H**) EHBP1L1 fluorescence (green). (**C**,**F**,**I**) Merged fluorescent channels (blue/red/green). (**A**–**C**) 28 dpp testis; (**D**–**F**) 35 dpp; (**G**–**I**) 42 dpp. Scale bars represent 20 μm, except for the insets, where they represent 10 μm. Striped arrowhead: SPG; arrow: I SPC; dotted arrow: rSPT.

EHBP1L1 localization together with PNA lectin, which marks the acrosome, was studied by immunofluorescence analysis on developing testis sections (7, 14, and 21 dpp, Figure 3; 28, 35 and 42 dpp, Figure 4).

As expected, no staining was evidenced at 7 (A–C) and 14 (D–F) dpp, where gonocytes (arrowhead; Figure 3C) and A/B SPG (striped arrowhead; Figure 3F) were the only germ cells comprising the developing seminiferous tubules. At 21 dpp (Figure 3G–I) EHBP1L1 signal appeared and localized in the perinuclear cytoplasm of I SPC (arrow; Figure 3I and inset), while it was still absent in SPG (striped arrowhead; Figure 2). From 28 dpp onward, the PNA lectin staining appeared, making it possible to follow the AB (Figure 4A–C).

At this stage, EHBP1L1 became localized in the perinuclear cytoplasm of SPC (arrow; Figure 4C) and rSPT (dotted arrow; Figure 4C and inset), with a more outlined appearance compared to that observed at 21 dpp, in which it was more diffused. SPG continued to be negative (striped arrowhead, Figure 4C). In 35 dpp tubules (Figure 4D–F), it was possible to identify the rSPT (dotted arrow; Figure 4F and inset), wherein acrosome began to enlarge and flatten on the nuclear surface, and an EHBP1L1 signal was detectable, with a perinuclear localization close to the forming acrosomal region. Then, at 42 dpp (Figure 4G–I), the protein retained its localization in SPC (arrow; Figure 4I) and rSPT (dotted arrow; Figure 4I and inset).

### 3.3. EHBP1L1 Localization in Adult Rat Testis and during AB

**Figure 5:**
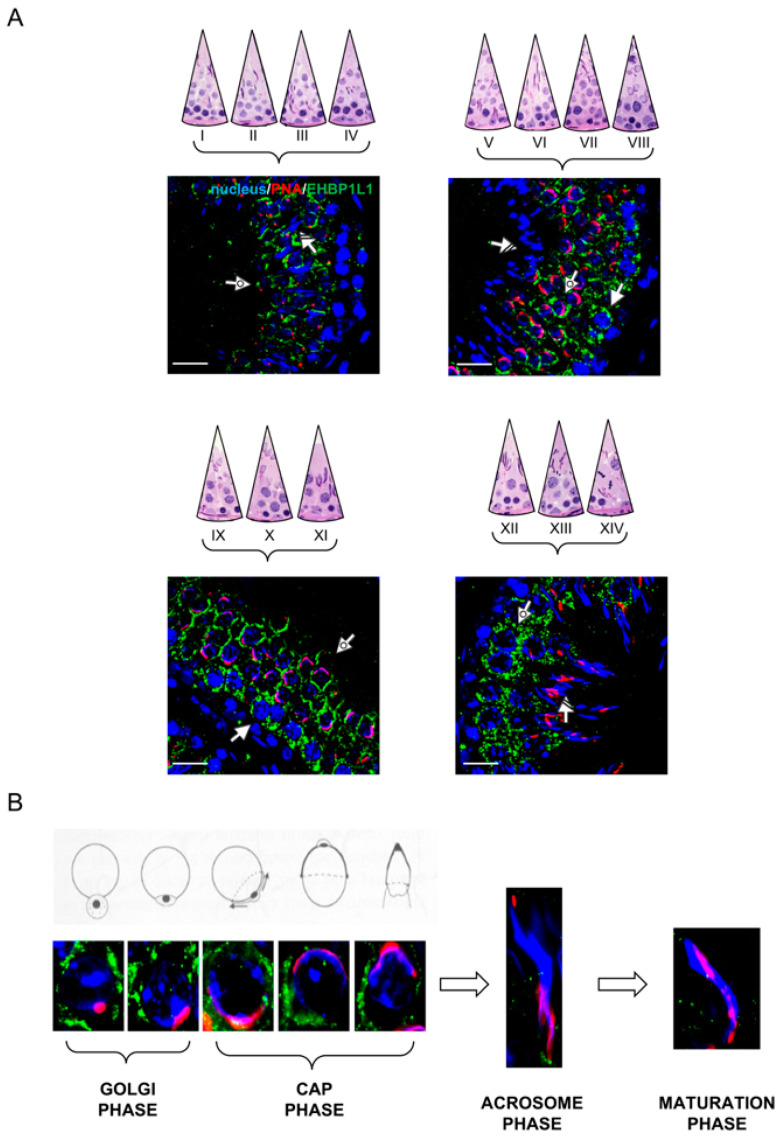
Localization of EHBP1L1 in adult rat testis (60 dpp). (**A**) EHBP1L1 localization in different stages of adult rat spermatogenesis. To better show the differences between them, the 14 stages comprising the whole spermatogenic cycle are arbitrarily sub-divided into four parts. Scale bars represent 20 μm. Arrow: I SPC; dotted arrow: rSPT; striped arrow: eSTP. (**B**) EHBP1L1 localization in SPT in comparison with a schematic representation of the different phases of acrosome biogenesis. In A and B merged images, the blue channel represents DAPI-fluorescent nuclear staining, the red channel represents PNA lectin acrosome staining, and the green channel represents EHBP1L1 fluorescent signal.

In rat adult testis (60 dpp), the spermatogenic cycle can be divided into 14 morphologically identifiable stages, characterized by the synchronous appearance and development of the same germ cells [21]. EHBP1L1 localization was analyzed during the spermatogenic cycle, and four representative images are showed in Figure 5A.

At 60 dpp, EHBP1L1 showed a similar localization pattern observed in the previous stage, being present predominantly in the perinuclear cytoplasm of SPC (arrow; Figure 5A) and rSPT (dotted arrow; Figure 5A), with a more pronounced intensity in stages XII-XIV and IX-XI, respectively. No positive signal was observed in eSPT (striped arrow; Figure 5A).

In Figure 5B, EHBP1L1 localization in the differentiating SPT is shown, associated with the acrosome system throughout the different phases of its biogenesis. In all the phases, the EHBP1L1 signal appeared quite intense and diffuse and started to decrease in the final part of the cap phase until its complete disappearance in the acrosome and maturation phases (Figure 5B).

### 3.4. Co-Localization of EHBP1L1 with Actin and Golgi during the First Wave of Rat Spermatogenesis

**Figure 6:**
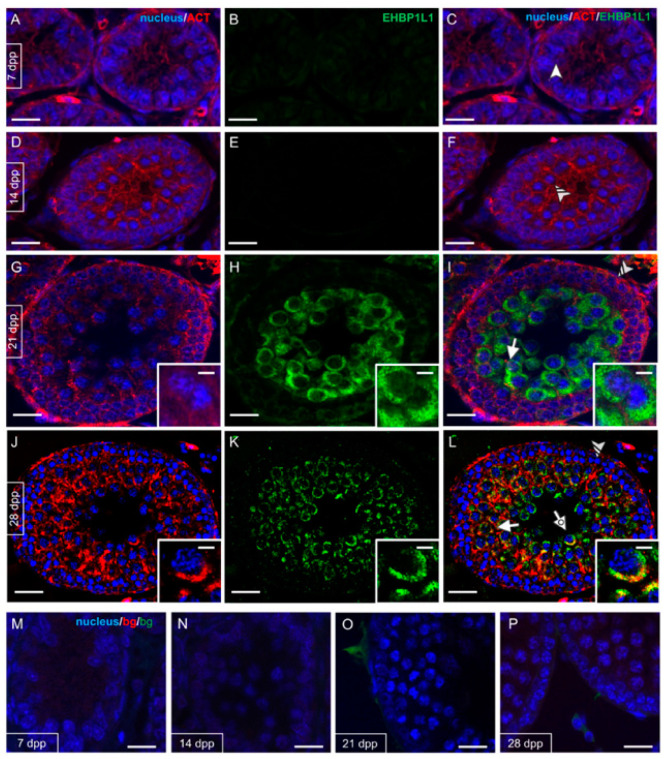
Co-localization of EHBP1L1 and ACTIN during the first wave of rat spermatogenesis (7–28 dpp). (**A**,**D**,**G**,**J**) DAPI-fluorescent nuclear staining (blue) and ACTIN staining (red). (**B**,**E**,**H**,**K**) EHBP1L1 fluorescence (green). (**C**,**F**,**I**,**L**) Merged fluorescent channels (blue/red/green). The intermediate yellow–orange tint indicates EHBP1L1 and ACTIN co-localization. (**A**–**C**) 7 dpp testis; (**D**–**F**) 14 dpp; (**G**–**I**) 21 dpp; (**J**–**L**) 28 dpp. (**M**–**P**) Negative controls for the same time points, obtained by omitting the primary antibodies. Scale bars represent 20 μm, except for the insets, where they represent 10 μm. Arrowhead: gonocytes; striped arrowhead: SPG; arrow: I SPC; dotted arrow: rSPT.

**Figure 7:**
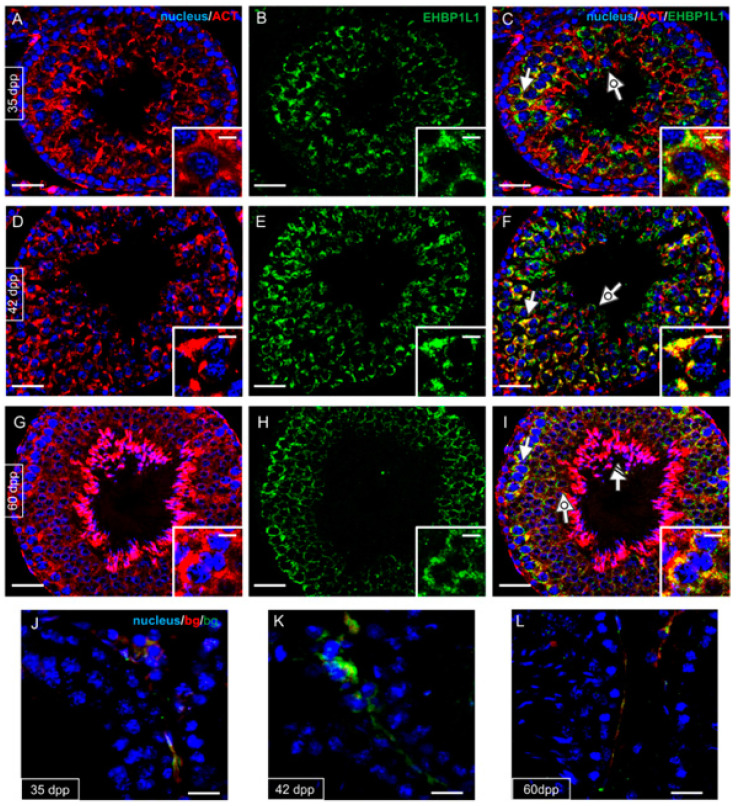
Co-localization of EHBP1L1 and ACTIN during the first wave of rat spermatogenesis (35–60 dpp). (**A**,**D**,**G**,**J**) DAPI-fluorescent nuclear staining (blue) and ACTIN staining (red). (**B**,**E**,**H**,**K**) EHBP1L1 fluorescence (green). (**C**,**F**,**I**,**L**) Merged fluorescent channels (blue/red/green). The intermediate yellow–orange tint indicates EHBP1L1 and ACTIN co-localization. (**A**–**C**) 35 dpp testis; (**D**–**F**) 42 dpp; (**G**–**I**) 60 dpp. (**J**–**L**) Negative controls for the same time points, obtained by omitting the primary antibodies. Scale bars represent 20 μm, except for the insets, where they represent 10 μm. Arrow: I SPC; dotted arrow: rSPT; striped arrow: eSPT.

Given EHBP1L1 association with actin microfilaments, the co-localization profile of the two proteins was performed at the same time point described above (Figure 6 and Figure 7).

Actin was detectable in gonocytes (arrowhead; Figure 6C) and SPG (striped arrowhead; Figure 6F) and then in meiotic SPC (arrow; Figure 6I and inset). At 28 dpp, the co-localization of EHBP1L1 appeared evident due to the intermediate yellow–orange tint in the perinuclear cytoplasm of SPC (arrow; Figure 6L) and rSPT (dotted arrow; Figure 6L), probably in the acrosomal region. In the following stages, ACTIN also localized in all stages and, to varying extents, by all cell types; its co-localization with EHBP1L1 was evident in the perinuclear cytoplasm of SPC (arrow; Figure 7C,F,I) and rSPT (dotted arrow; Figure 7C,F,I and insets). Finally, inside the adult testis, ACTIN localized in eSPZ (striped arrow, Figure 7I).

**Figure 8:**
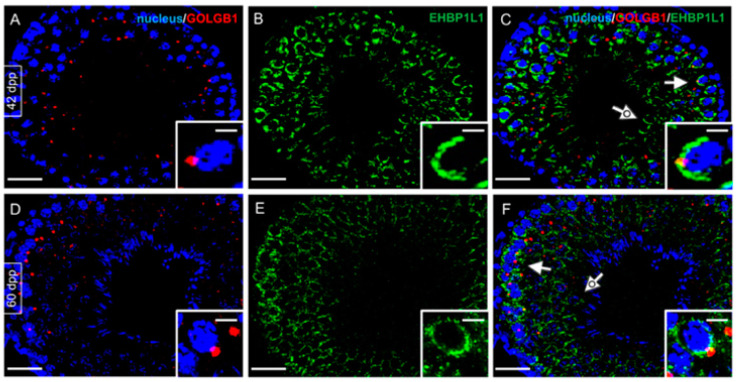
Co-localization of EHBP1L1 and GOLGB1 at 42 and 60 dpp. (**A**,**D**) DAPI-fluorescent nuclear staining (blue) and GOLGB1 staining (red). (**B**,**E**) EHBP1L1 fluorescence (green). (**C**,**F**) Merged fluorescent channels (blue/red/green). (**A**–**C**) 42 dpp testis; (**D**–**F**) 60 dpp. GOLGB1 staining, initially green, was modified into red to obtain the merged channels. Scale bars represent 20 μm, except for the insets, where they represent 10 μm. Arrow: I SPC; dotted arrow: rSPT; striped arrow: eSPT.

EHBP1L1 localization analysis was also extended using GOLGB1, a marker of the Golgi apparatus, in 42 dpp (Figure 8A–C), the stage in which the highest level of EHBP1L1 was detected, and in 60 dpp (Figure 8D–F), which represents the most mature stage wherein all the germ cells can be identified. As previously observed, EHBP1L1 retains its localization in the perinuclear cytoplasm of SPC (arrow; Figure 8C,F) and rSPT (dotted arrow; Figure 8C,F and insets) surrounding the Golgi region, which appears as a red dot. eSPT was negative for both proteins (asterisk, Figure 8F).

### 3.5. EHBP1L1 Analysis in Isolated Germ Cells and in Rat and Human SPZ

**Figure 9:**
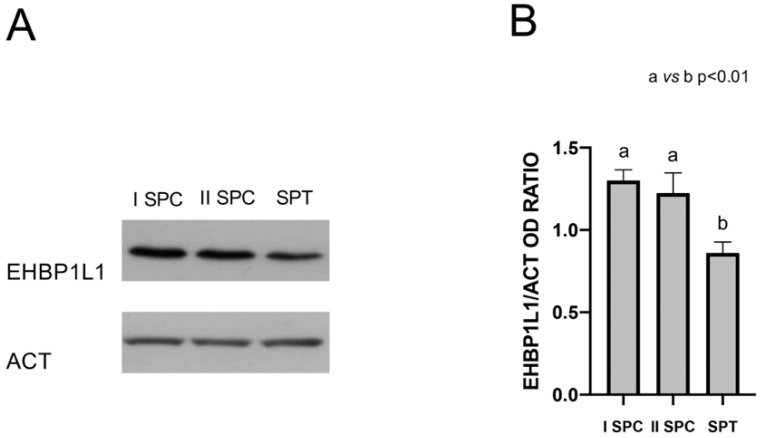
EHBP1L1 protein level in isolated germ cells. (**A**) Western blot analysis showing the expression of EHBP1L1 (164 kDa, top section) and ACTIN (42 kDa, bottom section), in I and II SPC and SPT enriched fractions. (**B**) Histogram showing the relative expression levels of EHBP1L1 in all the analyzed samples. Data were normalized with ACTIN and reported as EHBP1L1/ACT OD ratio. All data represent the mean ± SEM. a vs. b: *p* < 0.01. Western blot experiment was performed in triplicate.

To further confirm the presence of EHBP1L1 in the specific cell types observed with the immunofluorescence, a Western blot analysis was performed on the protein extract form I and II SPC and SPT enriched fractions obtained via an elutriation experiment (Figure 9).

As expected, we observed a single band of 164 kDa in all the three fractions (Figure 9A); moreover, the analysis of its relative protein level, reported as the EHBP1L1/ACT OD ratio (Figure 9B), revealed a lowest level in the SPT fraction, as compared to the other two (*p* < 0.01).

**Figure 10:**
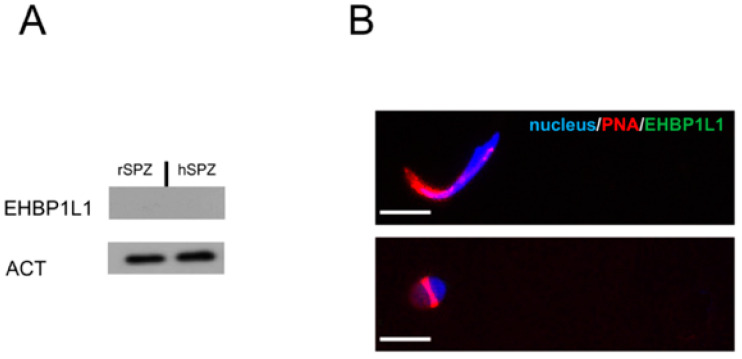
EHBP1L1 protein level and localization in rat and human SPZ. (**A**) Western blot analysis showing the expression of EHBP1L1 (164 kDa, top section) and ACTIN (42 kDa, bottom section) in rat (lane 1) and human (lane 2) SPZ. Each Western blot experiment was performed in triplicate. (**B**) Immunofluorescence analysis of EHBP1L1 in rat and human SPZ. Scale bars represent 10 μm.

Finally, to confirm the absence of EHBP1L1 in male gametes, a Western blot (Figure 10A) and immunofluorescence (Figure 10B) analysis were carried out on rat epididymal SPZ. Both the analyses revealed the total absence of the signal for EHBP1L1, indicating that they were eliminated from the SPZ prior to their spermiation.

To better clarify this point, since, in rats, spermiation and epididymal modifications do not allow for a clear observation of the cytoplasmic droplet, the analysis was extended to human ejaculated SPZ (Figure 9A,B), where the droplet is often visible [16,22]. To ascertain the presence of this structure in the analyzed samples, we performed an immunofluorescence staining using the anti-DAAM1 antibody, an actin-associated protein that, in a previous paper, we demonstrated that it was retained in the cytoplasmic droplet (data not shown) [23]. These analyses also confirmed the previous data; the EHBP1L1 signal was not detected in the gametes, considering that the peptide used to raise the antibody presents 100% of sequence identity with human EHBP1L1 isoforms 1 and 2 and 91% of sequence identity with rat EHBP1L1 isoforms (see Figure S1).

## 4. Discussion

It is well known that the production of good-quality gametes is essential for successful reproduction, which is the final goal of a species for its continuation. In addition, the morphological and molecular quality of gametes is a necessary aspect to ensure efficacious fertilization and correct embryo development [24]. Conversely, currently, infertility is one of the main concerns since it affects almost 200 million people worldwide, of which male infertility contributes to 50% [25]. Spermatogenesis, the proliferative and differentiative progression that leads to SPZ formation, is an extremely intricate and delicate process, which implies different phases and, due to its intrinsic complexity, many mistakes can happen because of exogenous (environmental pollutions) [26,27,28,29,30] and endogenous (genetic defects, anatomic abnormalities) [31] factors. Among the varying defects that can affect SPZ, influencing their quality, one of the clearer and most evident manifestations of male infertility is globo-theratozoospermia, namely, the presence of SPZ with an abnormal head, often characterized by a defect or total absence of the acrosome [7,32].

For this, it becomes particularly crucial to study the cellular and molecular mechanisms underlying spermatogenesis to eventually develop intervention strategies to improve gametic quality and, consequently, fertility [33]. One of the most peculiar events occurring during spermatogenesis is spermiogenesis, the cellular differentiation of rSPT first into eSPT and then into SPZ. AB occurs via four phases: (1) the Golgi phase (stages I-IV of the spermatogenic cycle), where the Golgi-derived proacrosomal vesicles move in a cytoskeleton-dependent route toward the nucleus to form a single granule; (2) the cap phase (stages V-XI of the spermatogenic cycle), where the granule enlarges and becomes a cap surrounding the nuclear envelope; (3) the acrosomal phase (stages XII-XIV of the spermatogenic cycle), where the acrosome condenses and begins to migrate over the ventral surface of the eSPT nucleus, gradually orientating toward the plasma membrane; (4) and finally, the maturation phase, where the acrosome spreads over the entire nuclear membrane while cytoplasmic components and several superfluous organelles (comprising vesicles, the Golgi apparatus, and ribosomes) are eliminated in the form of cytoplasmic droplets prior to spermiation [6,7,8].

In this particularly complex scenario, the fundamental role played by the cytoskeleton and its associated proteins should be highlighted, in which they not only help eSPT into their intimate morphological differentiation in SPZ [23,34,35], but they also drive the vesicular trafficking of proacrosomal granules during AB [6].

In this regard, we recently isolated a partial cDNA clone encoding for *EHBP1L1* [11]. We found that *EHBP1L1* was specifically expressed in I and II SPC, while it was completely absent in SPG, SPT and SPZ, leading us to hypothesize its role in AB. Herein, we further characterized EHBP1L1, analyzing its protein temporal and spatial expression during the first wave of rat spermatogenesis. Western blot and immunofluorescent analysis showed that the protein appeared at 21 dpp, the period where I SPC is produced, according to our previous paper [11]. In the NCBI database, there are four rat EHBP1L1 isoforms deposited that differ in length of their amino acid sequence and, consequently, in molecular weight. Results from our Western blot analyses revealed only one band of about 164 kDa, which allowed us to suggest that only one variant, likely EHBP1L1 isoform 2, is expressed in the rat testis, which also considers that all the other isoforms present a lower predicted nonglycosylated molecular weight. This suggestion is corroborated by the fact that, in our previous paper [11], the PCR experiment evidenced the same isoform; thus, we can assume that at least isoform 2 is expressed in the rat testis. Taking into account that all the isoforms possess the same conserved domains required by EHBP1L1 to regulate vesicular trafficking, the identification of the specific isoform involved in AB is not as relevant to prove the role of EHBP1L1 in this process.

Notoriously, SPCs (and their female equal) are the only cells that undergo meiosis; remarkably, proteins containing CH domains, such as EHBP1L1, have been associated with the formation of the actin contractile actomyosin ring [36,37,38]; thus, in SPC, EHBP1L1 may regulate cytoskeletal organization to permit proper cell division.

From 28 dpp, when rSPT appears (and the AB starts and lasts until the ending of the first spermatogenic wave), we found that the EHBP1L1 signal was also present in these cells. The immunofluorescent analysis highlighted that the protein co-localized in the perinuclear cytoplasm close to the acrosomal and Golgian regions with actin, its cytoskeletal partner. It has been demonstrated by Nakajo et at. [12] that polarized epithelial cells such as EHBP1L1, through the bivalent Mical/EHBP Rab binding (bMERB) and proline-rich domains present in its aminoacidic sequence, can interact with the small GTPases Rab8/10, Bin1/amphiphysin II, and dynamin, forming a multiprotein complex that regulates vesicular transport from the trans-Golgi network to the apical plasma membrane. Considering this assumption, as well as the fact that round and elongated SPT are highly specialized epithelial cells, EHBP1L1 may only be involved in AB, regulating vesicular trafficking, particularly during the Golgi and cap phases.

Our hypothesis is supported by the fact that *EHBP1L1* mRNA is expressed only in SPC, as previously demonstrated [11], and by the data reported in this paper, in which it is shown that the protein is also present in SPT, exhibiting a lower level of expression compared to those observed in I and II SPC. Immediately before the progressive transcriptional inactivation that occurs during spermiogenesis, two significant bursts of RNA synthesis occur in SPC and in rSPT, to be subsequently translated into proteins in differentiating SPT [39,40,41]. Thus, we assumed that EHBP1L1 expression starts in I and II SPC and that the presence of the protein only in differentiating SPT may be useful in sustaining the trafficking of the vesicles originating from the Golgi apparatus for the AB.

Finally, no positive signal for EHBP1L1 was observed in rat SPZ, neither in those present in the seminiferous epithelium, nor in those isolated from the epididymis. Once again, these data support our hypothesis; probably having concluded its role during AB, EHBP1L1 is removed in the mature SPZ prior to their release in the tubular lumen. This passage is of interest since, as above-mentioned, many organelles and cytoplasmic components are eliminated via the cytoplasmic droplet, forming a residual that is phagocytized by the adjacent Sertoli cell; thus, EHBP1L1 may follow the same fate. Since the cytoplasmic droplet is a morphological feature that is difficult to identify in rat SPZ, we extended the analysis to human SPZ, where the droplet is often found in ejaculated gametes, to easily compare EHBP1L1 final localization with that observed in rats. As expected, the Western blot analysis did not evidence EHBP1L1 expression in human SPZ, and this result was confirmed by the absence of any visible cytoplasmic droplets and due to the absence of any EHBP1L1 fluorescent signal.

## 5. Conclusions

In conclusion, this paper reports, for the first time, a putative role for EHBP1L1 in rat spermatogenesis. In particular, we found that during the first spermatogenic wave its expression started at 21 dpp, concomitantly to the appearance of I SPC, and lasted until the end. The protein specifically localized, together with actin microfilaments, in the perinuclear cytoplasm of meiotic and post-meiotic cells, close to the developing acrosome. These data, supported by our previous report indicating the I and II SPC as the cells expressing *EHBP1L1* mRNA, suggest that the relative protein may be involved in AB that is realized during spermiogenesis. Further confirmation came from the complete absence of the signal in rat and human mature SPZ, proving that the protein is needed in the previous stages of differentiation. Although these results are suggestive and fascinating, further studies are needed, not only to better clarify all the underlying molecular mechanisms of AB, but also to extend the analysis to humans, with the aim to use EHBP1L1 as a potential new marker of normal spermatogenesis.

## Data Availability

The data presented in this study will be made available upon request to the corresponding authors.

## Supplementary Materials

The following supporting information can be downloaded at: https://www.mdpi.com/article/10.3390/biomedicines10010181/s1, Figure S1: Blast analysis, obtained using the protein BLAST tool of NCBI website, of sequence similarity of the peptide used by Biorbyt to produce EHBP1L1 antibody with rat and human proteome sequence.
