# Supplementary material for: Differential Expression and Localization of EHBP1L1 during the First Wave of Rat Spermatogenesis Suggest Its Involvement in Acrosome Biogenesis
Source: Biomedicines. 2022 Jan 16;10(1):181. doi: 10.3390/biomedicines10010181 (PMC8773523; doi:10.3390/biomedicines10010181)
Supplement: Supplementary file 1 [file biomedicines-10-00181-s001.zip › biomedicines-1520446-supplementary.pdf]

A

IIENESKGQRKVLATAEVDLARHAGPVPVQVPVRLRLKPKSVKVVQAELSL
TLSGVLLREGRATDDDMQSLASLMSVKPSDVGNLDDFAESDEDEAHGPG

B

Description	Scientific Name	Max Score	Total Score	Query Cover	E value	Per. Ident	Acc. Len	Accession
EH domain-binding protein 1-like protein 1 isoform 4 [Rattus norvegicus]	Rattus norvegicus	184	184	100%	2e-55	91.00%	762	NP_001257634.1
EH domain-binding protein 1-like protein 1 isoform 1 [Rattus norvegicus]	Rattus norvegicus	184	184	100%	3e-55	91.00%	774	NP_001020288.2
EH domain-binding protein 1-like protein 1 isoform 2 [Rattus norvegicus]	Rattus norvegicus	184	184	100%	6e-55	91.00%	1699	NP_001123469.1
rCG47454, isoform CRA_b [Rattus norvegicus]	Rattus norvegicus	184	184	100%	6e-55	91.00%	877	EDM12523.1
EH domain-binding protein 1-like protein 1 isoform 3 [Rattus norvegicus]	Rattus norvegicus	183	183	100%	9e-55	91.00%	939	NP_001257633.1
EH domain-binding protein 1 [Rattus norvegicus]	Rattus norvegicus	90.5	90.5	85%	5e-22	58.14%	1202	NP_001292059.1
EH domain binding protein 1 (predicted) [Rattus norvegicus]	Rattus norvegicus	90.5	90.5	85%	6e-22	58.14%	1146	EDL97964.1

C

Description	Scientific Name	Max Score	Total Score	Query Cover	E value	Per. Ident	Acc. Len	Accession
EH domain-binding protein 1-like protein 1 isoform 2 [Homo sapiens]	Homo sapiens	199	199	100%	2e-60	100.00%	765	NP_001338016.1
EH domain-binding protein 1-like protein 1 isoform 1 [Homo sapiens]	Homo sapiens	201	201	100%	3e-60	100.00%	1523	NP_001092879.1
hCG23295 [Homo sapiens]	Homo sapiens	199	199	100%	8e-60	99.00%	1508	EAW74410.1
hypothetical protein [Homo sapiens]	Homo sapiens	199	199	100%	9e-60	99.00%	1510	CAD39093.1
FLJ00043 protein [Homo sapiens]	Homo sapiens	184	184	92%	2e-54	98.91%	1415	BAB15741.1
unknown [Homo sapiens]	Homo sapiens	104	104	85%	2e-28	56.98%	211	AAX82024.1
unnamed protein product [Homo sapiens]	Homo sapiens	104	104	85%	1e-27	56.98%	310	BAA91391.1
EH domain-binding protein 1 isoform 3 [Homo sapiens]	Homo sapiens	104	104	85%	2e-26	56.98%	1160	NP_001136087.1
EH domain-binding protein 1 isoform 5 [Homo sapiens]	Homo sapiens	104	104	85%	2e-26	56.98%	1195	NP_001341146.1
EH domain-binding protein 1 isoform 8 [Homo sapiens]	Homo sapiens	104	104	85%	2e-26	56.98%	878	NP_001341151.1
EH domain-binding protein 1 isoform 6 [Homo sapiens]	Homo sapiens	104	104	85%	2e-26	56.98%	1159	NP_001341148.1
unnamed protein product [Homo sapiens]	Homo sapiens	104	104	85%	2e-26	56.98%	1160	BAF85234.1
EH domain-binding protein 1 isoform 1 [Homo sapiens]	Homo sapiens	104	104	85%	2e-26	56.98%	1231	NP_001341141.1
EH domain-binding protein 1 isoform 2 [Homo sapiens]	Homo sapiens	104	104	85%	2e-26	56.98%	1196	NP_001136086.1
EH domain-binding protein 1 isoform 4 [Homo sapiens]	Homo sapiens	104	104	85%	2e-26	56.98%	1230	NP_001341144.1
EH domain-binding protein 1 isoform 7 [Homo sapiens]	Homo sapiens	104	104	85%	2e-26	56.98%	949	NP_001341150.1
NPF/calponin-like protein [Homo sapiens]	Homo sapiens	103	103	85%	5e-26	55.81%	1231	AAQ97141.1

Figure S1. Blast analysis, obtained using the protein BLAST tool of NCBI website, of sequence similarity of the peptide used by Biorbyt to produce EHBP1L1 antibody (A) with rat (B) and human (C) proteome sequence. The peptide, which is KLH conjugated synthetic peptide derived from human EHBP1L1:101-200/1523, has 91% of similarity with rat EHBP1L1 isoforms and 58% of similarity with EHBP1. Similarly, the peptide has 100% of identity with human EHBP1L1 isoforms and 57% of similarity with EHBP1.
